# HGF/c-Met signalling promotes Notch3 activation and human vascular smooth muscle cell osteogenic differentiation *in vitro*

**DOI:** 10.1016/j.atherosclerosis.2011.08.033

**Published:** 2011-12

**Authors:** Yiwen Liu, Tao Wang, Jianyun Yan, Naomi Jiagbogu, Daniëlle A.M. Heideman, Ann E. Canfield, M. Yvonne Alexander

**Affiliations:** a Cardiovascular Research Group, University of Manchester, UK; b Medical Genetics Research Group, University of Manchester, UK; c Wellcome Trust Centre for Cell-Matrix Research, University of Manchester, UK; d Department of Pathology, VU University Medical Centre, Amsterdam, The Netherlands

**Keywords:** Calcification, Hepatocyte growth factor, c-Met, NK4, Notch signalling, Vascular smooth muscle cells

## Abstract

**Objectives:**

Vascular calcification is a major clinical problem and elucidating the underlying mechanism is important to improve the prognosis of patients with cardiovascular disease. We aimed to elucidate the role and mechanism of action of Hepatocyte Growth Factor (HGF)/c-Met signalling in vascular calcification and establish whether blocking this pathway could prevent mineralisation of vascular smooth muscle cells (VSMCs) *in vitro*.

**Methods and results:**

We demonstrate increased HGF secretion and c-Met up-regulation and phosphorylation during VSMC osteogenic differentiation. Adenoviral-mediated over-expression of HGF (AdHGF) in VSMCs accelerated mineralisation, shown by alizarin red staining, and significantly increased ^45^Calcium incorporation (1.96 ± 0.54-fold [*P* < 0.05]) and alkaline phosphatase (ALP) activity (3.01 ± 0.8-fold [*P* < 0.05]) compared to controls. AdHGF also significantly elevated mRNA expression of bone-related proteins, Runx2, osteocalcin, BMP2 and osterix in VSMCs. AdHGF-accelerated mineralisation correlated with increased Akt phosphorylation, nuclear translocation of Notch3 intracellular domain (N3IC) and up-regulation of the Notch3 target protein, HES1. In contrast, adenoviral-mediated over-expression of the HGF antagonist, NK4, markedly attenuated VSMC mineralisation, and reduced c-Met phosphorylation, Akt activation and HES1 protein expression compared to AdHGF-treated cells. Furthermore, the Notch inhibitor, DAPT, attenuated N3IC nuclear translocation and AdHGF-induced mineralisation.

**Conclusion:**

We demonstrate HGF induces VSMC osteogenic differentiation *via* c-Met/Akt/Notch3 signalling, highlighting these pathways as potential targets for intervention of vascular calcification.

## 1 Introduction

The presence of vascular calcification is a severe clinical problem, and is prognostic for an increased risk of adverse cardiovascular outcomes [1]. It is now well-established that vascular calcification results from transformation of vascular smooth muscle cells (VSMCs) into osteo/chondrocytic-like cells in response to local micro-environmental cues, such as elevated calcium and phosphate levels, oxidative stress and several cytokines [2]. However, methods to attenuate vascular calcification *in vivo* have not yet been elucidated.

Hepatocyte growth factor (HGF) is a mesenchyme-derived cytokine with multiple biological effects on a wide variety of cells including mitogenic, motogenic, morphogenic, and anti-apoptotic activities. It can be inhibited by NK4, which consists of the N-terminal hairpin domain and the four kringle domains of the HGF α-chain [3], [4]. Serum HGF has been linked with progression of atherosclerosis [5], and localised expression of HGF and its receptor, c-Met, have been detected in atherosclerotic lesions [6]. HGF/c-Met signalling can also promote osteogenic differentiation of bone marrow stromal cells [7]. However, whether HGF/c-Met signalling regulates the osteogenic differentiation of VSMCs is unknown.

HGF/c-Met signalling operates through many downstream targets, including PI3K/AKT and Notch in myocardium [8]. The Notch family comprises four transmembrane receptor proteins (Notch 1–4). Cleavage of the receptor leads to release and nuclear translocation of the intracellular domain (NICD), activating downstream target genes, which play important roles in vascular development [9]. Recently, Notch1 was detected in calcified atherosclerotic plaque, but not in normal vessels [10]. Furthermore, over-expression of Notch1 NICD significantly increased matrix mineralisation in human aortic SMCs [10]. Although Notch3 is expressed in human adult smooth muscle cells [11], neither its expression in calcific lesions, nor its role in VSMC osteogenic transition have been studied.

This study shows for the first time that activation of HGF/c-Met signalling accelerates VSMC mineralisation, a process attenuated by the HGF antagonist NK4. HGF-accelerated mineralisation of VSMCs *in vitro* occurs with concomitant phosphorylation of Akt, activation of Notch3 and up-regulation of the Notch3 target transcription factor HES1. Finally, we demonstrate that N-[N-(3,5-Difluorophenacetyl-Lalanyl)]-S-phenylglycine t-Butyl Ester (DAPT), an inhibitor of Notch signalling, attenuates both mineralisation and AdHGF-accelerated VSMC mineralisation *in vitro*, supporting links between Notch signalling and vascular calcification.

## 2 Materials and methods

Further details are provided in the online supplement.

### 2.1 Tissue collection and human vascular smooth muscle cell culture

Approval from the Local Research Ethics Committee was granted for human tissue use, and procedures were in accordance with institutional guidelines. The investigation also conforms with the principles outlined in the Declaration of Helsinki. VSMC explantation was carried out as previously described [12], and the age and gender of the donors are listed in supplementary Table 1. Experiments were performed with three individual cell populations, thus providing biological triplicates. Human tibial VSMCs were cultured in SMC growth media (PromoCell) and used between passages 4 and 7. See online supplement for detailed culture conditions and adenoviral transduction.

### 2.2 Induction and determination of calcification

Mineralisation of VSMCs was induced using osteogenic media (see online supplement) Protocols for determining osteogenic differentiation and calcification, *i.e.* alkaline phosphatase assay, alizarin red staining and the calcium assay are described in the online supplement.

### 2.3 Hepatocyte growth factor enzyme-linked immunosorbent assay

The concentration of HGF in 48 h-conditioned media (50 μl) from confluent VSMCs grown in regular or osteogenic media was determined at specific time-points by ELISA (Quantikine HGF Immunoassay kit, R&D Systems), according to the manufacturer's instructions.

### 2.4 Quantitative RT-PCR

RNA was isolated from cells using TRI_ZOL_ (Invitrogen), followed by cDNA synthesis using Mu-MLV reverse transcriptase (Eurogentec). Quantitative PCR was performed in quadruplicate samples by a StepOne PCR system (Applied Biosystems) with SYBR Green (Eurogentec). Primers were purchased from Qiagen (supplement Table 2). Expression levels of target genes were corrected using 18S and calculated with the comparative cycle threshold (*C*_T_) method.

### 2.5 Western blot and immunoprecipitation analysis

Primary antibodies were used in western blotting to detect expression of HGF (sc-7949, 1:500), c-Met (sc-161, 1:500), Notch3 intracellular domain (M-134, 1:1000), HES1 (sc-25392, 1:1000), and β-tubulin (sc-5274, 1:500) (Santa Cruz); phosphorylated Akt (no.9271, 1:1000) and total Akt (no.9272, 1:1000) (Cell Signalling Technology Inc); osteoprotegerin (MAB8051, 1:500) (R&D Systems). The phosphorylation status of c-Met was analysed by immunoprecipitation and immunoblotting. See online supplement for further details.

### 2.6 Immunofluorescence

Cells were incubated with an antibody against the intracellular domain of Notch3 (M-134) followed by Alexa Fluor 488 anti-rabbit IgG (A11034, Invitrogen). Rabbit IgG was the negative control. Nuclei were counterstained with DAPI.

### 2.7 Statistical analysis

Data shown are representative of at least 3 independent experiments using cells from three different patients. Data are expressed as mean ± SD. Differences between groups were analysed with a Student's *t* test or ANOVA. *P* < 0.05 was considered statistically significant.

## 3 Results

### 3.1 HGF/c-Met signalling is activated in VSMCs undergoing osteogenesis

As previously reported [13], when confluent VSMCs are cultured in the presence of β-glycerophosphate (β-GP, 5 mmol/L) and elevated calcium (2.6 mmol/L) (osteogenic media), a calcified matrix is deposited, which stains positive with alizarin red. No mineralisation is detected in VSMCs cultured with regular growth media for 21 days (supplement Fig. 1).

**Fig. 1:**
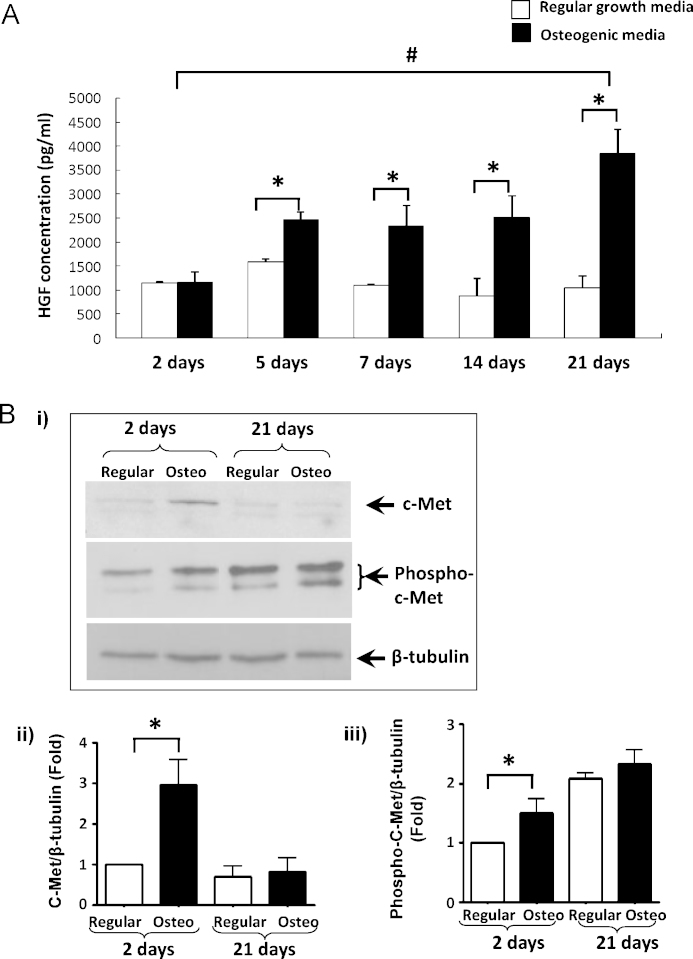
(A) ELISA showing HGF levels in conditioned medium from SMCs grown in osteogenic or regular media at specific time-points. *P < 0.05, osteogenic vs normal conditions on the same day; ^#^*P* < 0.05, day 21 vs day 2, in osteogenic conditions. (B) Western blot and densitometry analysis showing c-Met expression and phosphorylation in SMCs cultured in osteogenic and regular media.

To determine whether HGF expression is modulated during osteogenic differentiation of VSMCs, the level of HGF in VSMC conditioned medium was measured using an ELISA. HGF concentrations in conditioned media from cells cultured in regular growth media was 1154.6 ± 19.3 pg/ml, 1581.4 ± 65.1 pg/ml, 1099.2 ± 36.2 pg/ml, 884.6 ± 361.7 pg/ml, 1040.1 ± 258.7 pg/ml, respectively, at day 2, 5, 7, 14, and 21. Significantly increased HGF levels were detected in VSMCs cultured in osteogenic media (2468.7 ± 156.7 pg/ml, 2324.0.6 ± 436.4 pg/ml, 2509.7 ± 453.3 pg/ml, 3871.7 ± 416.9 pg/ml) on days 5, 7, 14, and 21, respectively (Fig. 1A). There was no significant difference between regular and osteogenic media on day 2.

The expression and activation of c-Met in VSMCs cultured in regular or osteogenic media were also determined. Western blot analysis demonstrated an increase in c-Met expression (3.0 ± 0.6-fold, *P* < 0.01) and phosphorylation (1.5 ± 0.2-fold, *P* < 0.05) as early as day 2 in VSMCs cultured in osteogenic media, compared to cells in regular media (Fig. 1B). However, this increase was not sustained and by day 21, c-Met expression and phosphorylation was similar in cells grown in both regular and osteogenic media. β-Tubulin shows protein loading.

### 3.2 AdHGF accelerates VSMC osteogenic differentiation

**Fig. 2:**
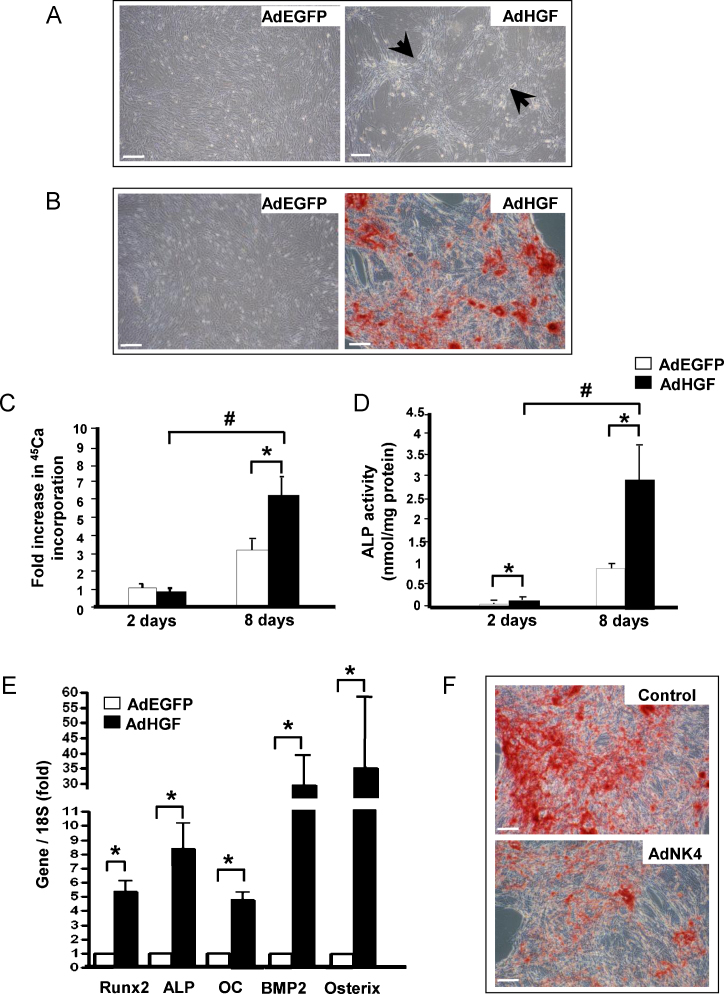
(A) VSMCs infected with AdEGFP or AdHGF and cultured in regular media for 21 days showed different growth patterns. (B) Alizarin red staining shows accelerated VSMC mineralisation in osteogenic media at day 8 in AdHGF-infected SMCs vs. AdEGFP-infected SMCs. Bar = 200 μm. (C) ^45^Ca accumulation and (D) ALP activity in VSMCs infected with AdEGFP and AdHGF and grown in osteogenic media. (*n* = 3) *P < 0.05 AdEGFP vs AdHGF-infected cells. ^#^*P* < 0.005 day 2 vs. day 8. (E) Expression of bone-related genes evaluated 2 days after infection by quantitative PCR. (*n* = 4) *P < 0.05 AdEGFP vs AdHGF. (F) Representative photomicrographs of AdNK4-infected and control VSMCs cultured in osteogenic media for 21 days (Control) and stained with Alizarin red shows VSMC mineralisation was attenuated in AdNK4-infected cells compared to controls. Bar = 200 μm.

To investigate the effects of activated HGF/c-Met signalling on VSMC mineralisation, recombinant adenovirus was used to over-express HGF in VSMCs *in vitro*; AdEGFP was used as a control. More than 80% of VSMCs were infected without evidence of cell toxicity (supplement Fig. 2). When VSMCs were maintained in regular growth media for 21 days, marked aggregation and retraction of HGF-over-expressing cells was noted (Fig. 2A, arrows). In contrast, this effect was absent in cells transduced with control AdEGFP virus (Fig. 2A) or non-infected controls (not shown). Furthermore, AdHGF-infected VSMCs, cultured in osteogenic media for 8 days showed accelerated matrix mineralisation; no mineral was detected in control AdEGFP-infected VSMC at this time-point (Fig. 2B).

Mineralisation was quantified by determining levels of ^45^Ca incorporation into the cell layer. The results (Fig. 2C) demonstrate calcium incorporation was significantly enhanced at day 8 (1.96 ± 0.55-fold, *P* < 0.05) in cells over-expressing HGF, compared to controls. ALP activity, (an early marker of osteogenic differentiation) was also increased 3.01 ± 0.80-fold (*P* < 0.05) in AdHGF-infected VSMCs compared to AdEGFP-infected controls after 8 days in culture (Fig. 2D). Furthermore, HGF over-expression significantly up-regulated mRNA levels of bone-related proteins, namely Runx2, ALP, Osteocalcin (OC), BMP2, and Osterix (Fig. 2E). Next, VSMCs were infected with AdNK4 to inhibit endogenous HGF/c-Met signalling and alizarin red staining demonstrates a marked attenuation of mineralisation in NK4-infected cells, vs AdEGFP-infected control cells grown in osteogenic media for 21 days (Fig. 2F).

### 3.3 Over-expression of HGF activates Akt phosphorylation in VSMCs

**Fig. 3:**
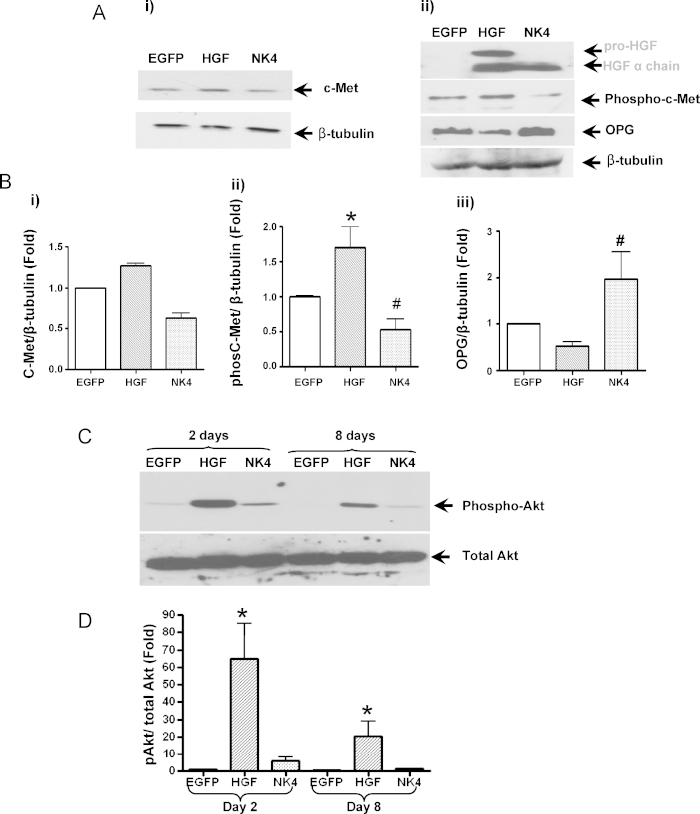
(A) and (B) Western blot and densitometry analysis show over-expression of HGF significantly increased phosphorylation of c-Met, but little effect on c-Met expression (Ai). In contrast, NK4 over-expression down-regulated c-Met phosphorylation (Aii & Bii). OPG expression was reduced in AdHGF-infected cells but increased in NK4-infected cells, compared to AdEGFP-infected cells (Aii & Biii). (C) Over-expression of HGF up-regulated phosphorylation of Akt at days 2 and 8 (*P < 0.01, ^#^*P* < 0.05, *n* = 3).

Since regulation and execution of HGF signalling is dependent on phosphorylation of c-Met, this was investigated in VSMCs after AdHGF-, AdNK4- and AdEGFP-infection (Fig. 3Ai & ii) and quantified by densitometry using β-tubulin as a protein loading control (Fig. 3B). Two days after infection, no change in c-Met expression was apparent (Fig. 3Ai & Bi) whilst a 1.7 ± 0.3-fold (*P* < 0.01) increase in c-Met phosphorylation was detected in HGF-over-expressing cells compared to controls (Fig. 3Aii & Bii). Of note, c-Met phosphorylation was reduced in NK4-over-expressing cells (0.5 ± 0.2-fold, *P* < 0.05) compared to control-infected cells (Fig. 3A and B). On day 8, c-Met expression and phosphorylation were comparable in all samples (supplement Fig. 3A and B). HGF over-expression also reduced the expression of the calcification inhibitor, osteoprotegerin (OPG), whereas AdNK4 up-regulated OPG expression (2.0 ± 0.6-fold, *P* < 0.05).

PI3K/Akt signalling is activated by phospho-c-Met [14]. Therefore, we examined Akt phosphorylation in VSMC infected with AdHGF, AdNK4 or control virus and cultured in osteogenic media. Over-expression of HGF markedly induced Akt phosphorylation at day 2 (65 ± 35-fold, *P* < 0.01), and although AdNK4 over-expression in VSMCs for 2 days showed elevated Akt phosphorylation compared to control cells, it was markedly reduced compared to cells induced to over-express HGF (Fig. 3C & D). By day 8, when AdHGF-infected VSMCs deposited a mineralised matrix, Akt phosphorylation was still elevated (20.5 ± 14.5-fold, *P* < 0.01), although it was reduced compared to 2 days following infection. No detectable Akt phosphorylation was observed in VSMCs infected with control virus (Fig. 3C & D). Low levels of Akt phosphorylation were still apparent in VSMCs 8 days after infection with AdNK4.

### 3.4 Over-expression of HGF activates notch3 signalling

**Fig. 4:**
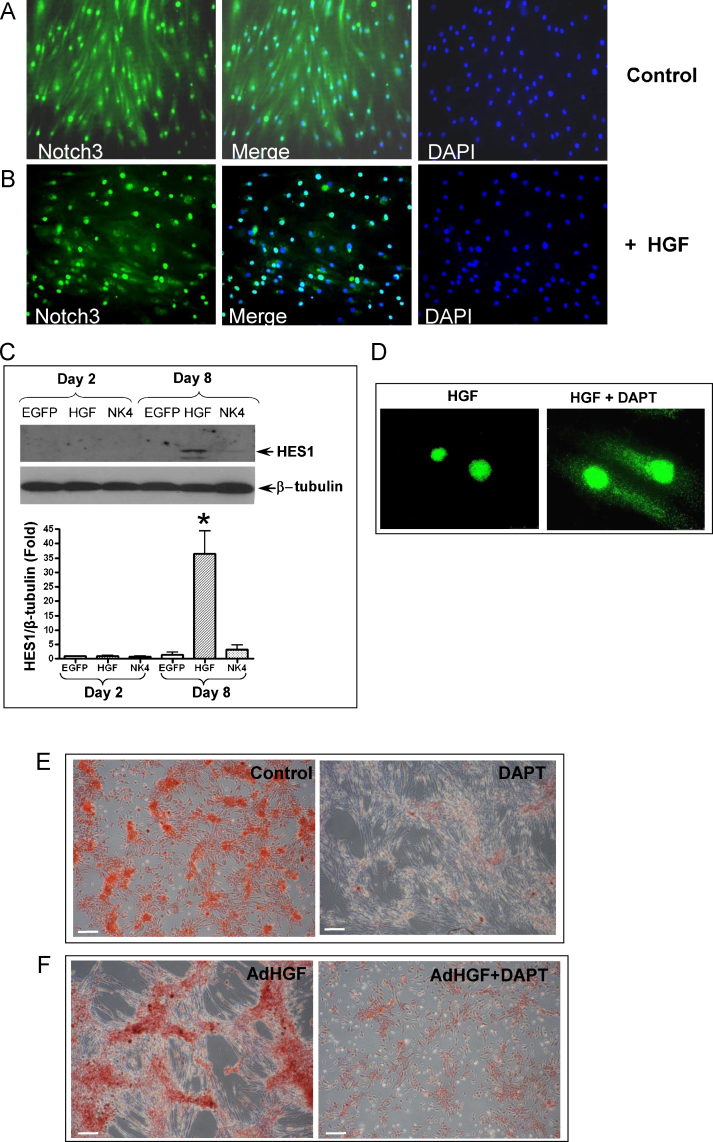
Representative micrographs show localisation of active Notch3 in SMCs infected with control AdEGFP (A) or AdHGF (B). (C) Western blotting show HES1 expression and β-tubulin expression as a loading control. *P < 0.01. (D) Localisation of Notch3 in VSMCs infected with AdHGF in the presence or absence of DAPT (1 μmol/L). (E) Alizarin red staining showed DAPT reduced mineralisation in VSMC cultured in osteogenic media for 21 days and (F) in SMCs infected with AdHGF and cultured in osteogenic media for 8 days (*n* = 3). Bar in *A* & *B* = 100 μm; *D* = 25 μm, *E* & *F* = 200 μm.

Notch3 is expressed in human adult smooth muscle cells [14], [15], but its role in VSMC osteogenic differentiation has not been investigated. Therefore, we interrogated Notch3 signalling during HGF-induced osteogenic differentiation of VSMCs. Notch3 was detected in both the cytoplasm and nuclei in control VSMCs grown in osteogenic media at day 2 (Fig. 4A), and in cells infected with AdBgl, an empty control virus (data not shown), indicating a low level of basal Notch3 activation in VSMCs.

However, 8 days after AdHGF infection of VSMCs, Notch3 was mainly localised in the nuclei, suggesting HGF over-expression triggers Notch3 activation (Fig. 4B). Validation of HGF-activation of Notch-3 comes from the finding that a significant increase in expression of the Notch3 downstream target protein HES1 was apparent 8 days after HGF over-expression (36 ± 8-fold, *P* < 0.01), whilst AdNK4 had an insignificant effect on HES1 expression (Fig. 4C). It is noteworthy that neither the Notch3 nuclear translocation nor the HES1 upregulation was observed after 2 days treatment, unlike the dramatic activation of PI3K/Akt signalling, suggesting that Notch3 signalling might act as a downstream target of the HGF/c-Met-PI3K/Akt axis.

Next, AdHGF-infected with VSMCs were treated with DAPT, a γ-secretase inhibitor that prevents N3IC release. We demonstrate that DAPT attenuates HGF-induced nuclear translocation of Notch3 (Fig. 4D). To establish whether DAPT-inhibition of Notch signalling would also modulate mineral deposition by VSMCs, cells were cultured in osteogenic media for 21 days in the presence or absence of DAPT. Alizarin red staining demonstrates that inhibition of Notch signalling by DAPT markedly attenuated mineral deposition by VSMCs (Fig. 4E). AdHGF-accelerated mineralisation was also reduced by DAPT (Fig. 4F), suggesting that Notch signalling is essential for HGF-induced VSMC osteogenic differentiation.

## 4 Discussion

**Fig. 5:**
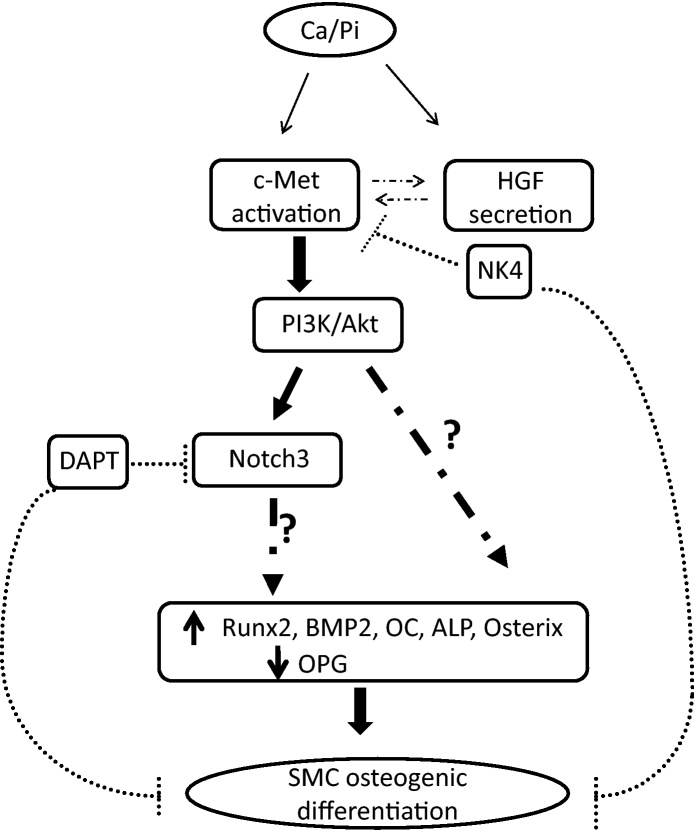
Model showing elevated calcium and/or phosphate up-regulate HGF secretion and c-Met activation in conjunction with activation of the PI3K/Akt signalling pathway, leading to SMC mineralisation, which can be inhibited by AdNK4 treatment. Signalling molecules and osteogenic transcription factors downstream of PI3K/Akt were shown to be involved in HGF/c-Met-induced mineralisation. We also show HGF/c-Met-induced Notch3 activation and using the Notch inhibitor, DAPT, we demonstrate attenuation of calcification suggesting Notch3 signalling involvement in this process. The dotted arrows between c-Met activation and HGF secretion indicate other factors could be associated with c-Met receptor and HGF ligand interaction.

In the vasculature, HGF/c-Met signalling has been associated with endothelial dysfunction and angiogenesis [16], and more recently with atherosclerosis [6], [17]. We demonstrate that activation of HGF/c-Met signalling could be an early response to certain pathological stimuli, such as elevated Ca and P, which triggers vascular calcification, thereby inducing VSMC osteogenic differentiation *via* activation of Akt and Notch3 signalling *in vitro*. We also show potential for inhibition of this process by either NK4 or DAPT (Fig. 5).

We demonstrate increased c-Met expression and phosphorylation in VSMCs 2 days after culture in osteogenic medium, which precedes the up-regulation of HGF secretion detected at day 5, suggesting that, under these conditions, a positive autocrine loop may exist between c-Met activation and HGF expression. It is possible that the c-Met receptor is up-regulated by the elevated calcium or phosphate conditions in the osteogenic media, thus leading to the apparent increased c-Met phosphorylation and immediate activation of Akt, with Notch3 activation at a later time-point. Elevated HGF expression also activated c-Met-PI3K/Akt-Notch3 signalling in VSMCs. Our results showed that HES1 expression was up-regulated 8 days after HGF over-expression, whereas HGF-induced c-Met activation was comparable to the control group at this time-point, suggesting that HGF/c-Met signalling could be negatively regulated by Notch3/HES1 in VSMCs; a finding consistent with a previous study showing negative feedback between HES1 and c-Met expression [18].

Previous reports have suggested that mesenchymal stem cells grow and organise into distinct patterns when they undergo osteogenic differentiation *in vitro* [19]. The present study demonstrates that VSMCs infected with AdHGF, aggregated together and retracted away from each other. In osteogenic conditions, AdHGF up-regulated mRNA levels of several bone-related proteins, namely, Runx2, ALP, OC, and BMP2. BMP2 is a potent inducer and morphogen in the process of SMC osteogenic differentiation. Therefore, our results indicate that HGF may either act as a novel morphogen in the early differentiation process of VSMCs or may facilitate VSMC differentiation *via* the BMP2 pathway. This suggestion is in accordance with Hossain et al. [7] who demonstrated that HGF enhances MC3T3-E1 pre-osteoblast differentiation by increasing Runx2, ALP, and OC mRNA expression. In addition, D’Ippolito et al. [20] demonstrated that when HGF was coupled with vitamin D treatment, it significantly increased ALP expression in bone marrow stromal cells, but mineralisation was only detected in the presence of HGF and 1,25-didroxy Vitamin D_3_ and not in the presence of HGF alone. Despite the fact that cultured VSMCs may undergo certain phenotypic changes *in vitro*, this is still a well-established *in vitro* model of vascular calcification. Therefore, the results from Hossain et al. [7] and D’Ippolito et al. [20], taken together with our studies demonstrating (i) mineralisation is induced by HGF only in the presence of high calcium and phosphate and not in regular growth media, and (ii) that mineralisation can be attenuated by AdNK4, suggest that under specific micro-environmental conditions, such as hypercalcemia or hyperphosphatemia, HGF could play an important role at several stages of vascular calcification.

Since c-Met is a transmembrane receptor tyrosine kinase, downstream events following HGF/c-Met binding were investigated. We show that Akt phosphorylation was increased at the 2-day time-point following HGF-over-expression and less so by NK4. Akt phosphorylation was undetectable in VSMCs infected with control virus and cultured in osteogenic conditions for 8 days, suggesting that early activation of Akt signalling may be involved in HGF-induced osteogenic differentiation of SMCs *in vitro*, and there is a possible positive-feedback between HGF and c-Met. The data from our study support previous studies which also show Akt activation during BMP-2-induced osteoblast differentiation of 2T3 cells [21], and also in bone development [22].

*In vivo* studies have shown that Notch3^−/−^ vessels lose their arterial phenotype, indicating that Notch3 is crucial for SMC phenotypic differentiation [23]. Since we have shown attenuation of mineralisation by both NK4 and DAPT, we speculate that HGF/c-Met-Notch3 axis could play an important role in the development of vascular calcification. Our data showing HGF/c-Met/Notch3 signalling in VSMCs support and extend the findings that NICD-expressing human mesenchymal stem cells undergo enhanced osteogenic differentiation [24], and also that HGF-treatment of cardiomyocytes up-regulates the Notch effector HES1 *via* PI3K/Akt signalling[8], suggesting an outside-in signal transduction cascade extending from HGF/c-Met to Notch.

In summary, this is the first report demonstrating that HGF/c-Met-PI3K/Akt-Notch3 signalling can regulate vascular calcification. Future studies using animal models will generate data that could have implications for the development of new targets for potential therapeutic intervention of this devastating pathology.

## Funding

This study was supported by The Universities UK and The University of Manchester as an ‘Overseas Research Studentship’ awarded to YL.

## Conflict of interest

None.
