# Supplementary material for: HGF/c-Met signalling promotes Notch3 activation and human vascular smooth muscle cell osteogenic differentiation in vitro
Source: Atherosclerosis. 2011 Dec;219(2):440–7. doi: 10.1016/j.atherosclerosis.2011.08.033 (PMC3925803; doi:10.1016/j.atherosclerosis.2011.08.033)
Supplement: Supplementary file 1 [file mmc1.doc]

**Liu *et al.* Over expression of HGF accelerates vascular calcification**

**Online Supplementary Material**

**Expanded Materials and Methods**

**Tissue Collection and Human Vascular Smooth Muscle Cell Culture**

Human tibial arterial segments, obtained from amputated limbs, were placed in saline and kept at 4°C until use (within 1-3 h). The muscle layer was dissected free from the surrounding mucosa, cut into small pieces <3 mm^3^, the luminal side of the explants was carefully placed in 6-well plates, in contact with the culture dish (6 to 10 tissue pieces per well) [1]. Cells were initially explanted using Bullet-2 (PromoCell, containing 5% FBS, 1.8 mmol/L CaCl_2_, bFGF (2.0 ng/ml), EGF (0.5 ng/ml), Insulin (5.0 μg/ml). Explanted tissue pieces were removed 1 week after the first SMCs appeared. When the cells had migrated from the explants, they were collected and maintained in regular growth media (Dulbecco’s Modified Eagle’s Medium (DMEM) (high glucose, 4.5 g/L) containing 5% FBS, 10 mmol/L sodium pyruvate, 1.8 mmol/L CaCl_2_, 100 U/mL penicillin, 100 mg/mL streptomycin. To confirm that the cells isolated were smooth muscle cells, immunofluorescence staining using antibodies against α-smooth muscle actin (anti-α-SMA, Sigma; positive) and von Willebrand factor (anti-vWF, DAKO; negative) was performed as described previously [2]. All experiments were performed using three different populations of cells obtained from three different patient tissue samples.

**Adenoviral Transduction**

VSMCs, at 80% confluency, were infected with recombinant adenovirus (Ad) using a multiplicity of infection (MOI) of 100 particles/cell. Infection was performed as described previously [3]. Viral vectors used were as follows: i) AdHGF, expressing full-length human HGF cDNA (provided by the Vector Core, Gene Therapy Program, Dept of Medicine at the University of Pennsylvania), ii) AdNK4, generated using the N-terminal hairpin domain and the four kringle domains of the α chain of HGF [4], iii) AdEGFP, expressing enhanced green fluorescence protein, to assess virus infection efficiency and iv) AdBgl as an empty control virus (both generated by the Gene Transfer Core, University of Iowa, USA under the direction of Dr. Beverly Davidson).

**Induction and Determination of Calcification**

Mineralization of VSMCs was induced using a modification of a method described by Reynolds *et al*. [5]. Briefly, VSMCs were seeded at a density of 4X10^4^ cells/cm^2^ and cultured in regular growth media until they reached 80% confluence. At that time, media was switched to osteogenic media, *i.e.,* regular growth medium containing 2.6 mmol/L CaCl_2_, and 5 mmol/L β-glycerophosphate and cultured for up to 21 days, when mineralization was apparent. Controls were maintained in DMEM containing 1.8 mmol/L CaCl_2_ without β-glycerophosphate. When N-[N-(3,5-Difluorophenacetyl-Lalanyl)]-S-phenylglycine t-Butyl Ester (DAPT) (#D5942 Sigma Aldrich) (1 μmol/L) was used to supplement medium, it was added when cells were changed to osteogenic media and was added each time the media was replaced, as were vehicle controls.

**i) Alizarin Red Stain**

Cells were fixed with 4% formaldehyde for 10 minutes and washed, followed by an incubation with alizarin red (2%, wt/vol, pH 4.2) for 5 minutes, and a final rinse with distilled water (pH 7.0) for an hour. Positive staining is red/orange.

**ii) Alkaline Phosphatase Assay**

Proteins were extracted from hSMC lysates by freeze-thawing the cells in 0.05% Triton X-100 in PBS. Total cellular proteins were quantified using a bicinchoninic acid (BCA) protein assay (Pierce Biotechnology). Total protein (15 μg) was assayed for alkaline phosphatase (ALP) activity as previously described^2^ and results are presented as nmol of nitrophenol formed per μg of cellular protein per minute. The data are from three experiments and are shown as the mean ± S.D.

**iii) Calcium Assay**

^45^Ca deposition was assayed as previously described.^2^ Briefly, VSMCs were seeded at a density of 4x10^4^ cells/cm^2^ in 6-well plates and when 80% confluence was reached (usually 24h after seeding), cells were infected with AdEGFP or AdHGF and cultured in osteogenic media. The media was supplemented with 0.5 μCi/ml of ^45^CaCl_2_ 48hrs prior to harvesting. The cells were collected in PBS (0.3 mL), at the appropriate time-point, placed into vials containing perchloric acid (0.2 mL) with 3% hydrogen peroxide (0.3 mL), and incubated at 80°C for 1 hour. Ethylene glycol monoethyl ether (1.6 mL) was added to the samples and a 1 ml aliquot was removed, diluted with Ecosint (1:5). Radioactivity was quantified by scintillation counting using a TRI-CARB 2000CA liquid scintillation counter.

**Western Blot Analysis and Immunoprecipitation**

Cell lysates (25 ug) were prepared and analyzed by western blotting as described [3]. Briefly, cell lysates were prepared using lysis buffer (20 mmol/L Tris-HCl pH 8.0; 2 mmol/L EDTA; 150 mmol/L sodium chloride; 1% Nonidet P-40) containing the following cocktail of protease and phosphatase inhibitors; 1 mmol/L sodium orthovanadate, 1 mmol/L phenylmethlysulfonylflouride, 1 mmol/L sodium pyrophosphate, 0.7 mg/ml pepstatin A, 1 mg/ml leupeptin. Samples were separated by 10% SDS-polyacrylamide gel electrophoresis (SDS/PAGE) and proteins were transferred to nitrocellulose membrane (Bio-Rad) as described [3]. Membranes were incubated overnight at 4°C, with specific antibodies as detailed in the text. Immunodetection was performed using an Enhanced Chemiluminescence Kit (ECL™ Plus, GE Healthcare) and autoradiography. Membranes were re-probed following submersion in 0.2 mol/L sodium hydroxide for 5 min at room temperature.

For immunoprecipitation, a BCA assay was performed on the cell lysates to quantify the total amount of protein used in each group. Equal amounts of protein were incubated with 20μL agarose-conjugated mouse anti-phospho-tyrosine antibodies (AGM1676, R&D Systems) for 16 hours at 4°C. The immunocomplexes were collected by centrifugation, washed and resuspended in 20μL of gel sample buffer (0.0625M Tris/HCl pH 6.8, 2% (w/v) β-mercaptoethanol) before being separated by electrophoresis in 10% SDS-polyacrylamide gels and analyzed by immunoblotting with anti-c-Met antibody. Agarose-conjugated anti-mouse IgGs served as controls for the immunoprecipitation.

**Immunofluorescence**

Human SMCs were fixed with acetone/methanol (1:1) for 10 minutes at -20°C. After permeabilization with 0.1% Triton x-100 in PBS for 5 minutes at RT, cells were blocked in 10% donkey serum/1% bovine serum albumin (BSA)/PBS for 1 hour at room temperature. Cells were incubated with primary Ab (Notch3 (M-134), 1:50 sc-5593) diluted in blocking buffer, at 4°C overnight, and donkey secondary antibodies conjugated to Alexa 488 (1:200, Molecular Probes) were used. Rabbit IgG was the negative control, and it gave negative staining (data not shown). Nuclei were counterstained with DAPI.

**References**

1. Campbell JH, Campbell GR. Culture techniques and their applications to studies of vascular smooth muscle. Clin Sci (Lond) 1993;85:501-513.

2. Kirton JP, Wilkinson FL, Canfield AE, Alexander MY. Dexamethasone downregulates calcification-inhibitor molecules and accelerates osteogenic differentiation of vascular pericytes: implications for vascular calcification. Circ Res 2006;98:1264-1272.

3. Collett GD, Sage AP, Kirton JP, Alexander MY, et al. Axl/phosphatidylinositol 3-kinase signaling inhibits mineral deposition by vascular smooth muscle cells. Circ Res 2007;100:502-509.

4. Heideman DA, van B, V, Bloemena E, et al. Suppression of tumor growth, invasion and angiogenesis of human gastric cancer by adenovirus-mediated expression of NK4. J Gene Med 2004;6:317-327.

5. Reynolds JL, Joannides AJ, Skepper JN, McNair R, Schurgers LJ, Proudfoot D, Jahnen-Dechent W, Weissberg PL, Shanahan CM. Human vascular smooth muscle cells undergo vesicle-mediated calcification in response to changes in extracellular calcium and phosphate concentrations: a potential mechanism for accelerated vascular calcification in ESRD. J Am Soc Nephrol 2004;15:2857-2867.

**Legends to Supplementary Figures**

**Suppl Figure I.** Osteogenic media induces human VSMC mineralization. Representative phase-contrast images from three independent experiments of VSMCs grown in regular media (i) or osteogenic growth media (ii) for 21 days, and stained with alizarin red. Mineral is deposited by VSMCs cultured under osteogenic conditions. Bar=200μm.

**Suppl Figure 2.** Dose-response of adenoviral MOI in human SMCs infected with EGFP adenovirus.Human VSMCs cultured in 6-well plates in SMC growth media were grown to 80% confluency and infected with increasing MOIs of AdEGFP. Expression of EGFP within cells correlated with an increasing MOI used. More than 80% infection efficiency was observed within cells at MOI of 100. Bar= 500 μm.

**Suppl Figure 3.** Regulation of c-Met phosphorylation by AdHGF and AdNK4.

Human VSMCs were either infected with AdEGFP or AdHGF, or AdNK4. After 8 days, cell lysates were prepared from each group of cells. Western blot and densitometry analysis shows no apparent change in c-Met expression (A) or phosphorylation at the 8-day time-point (B). n=3

**Suppl Table 1.** Characteristics of patients used in this study. Tibial arteries (TA) were obtained when patients underwent a lower limb amputation. F=female, M=male. Ethics approval ensured patient anonymity.

**Suppl Table 2.** Qiagen primers used for quantitative PCR.
