# Supplementary material for: HGF/c-Met signalling promotes Notch3 activation and human vascular smooth muscle cell osteogenic differentiation in vitro
Source: Atherosclerosis. 2011 Dec;219(2):440–7. doi: 10.1016/j.atherosclerosis.2011.08.033 (PMC3925803; doi:10.1016/j.atherosclerosis.2011.08.033)
Supplement: Supplementary file 2 [file mmc2.ppt]

## Slide 1
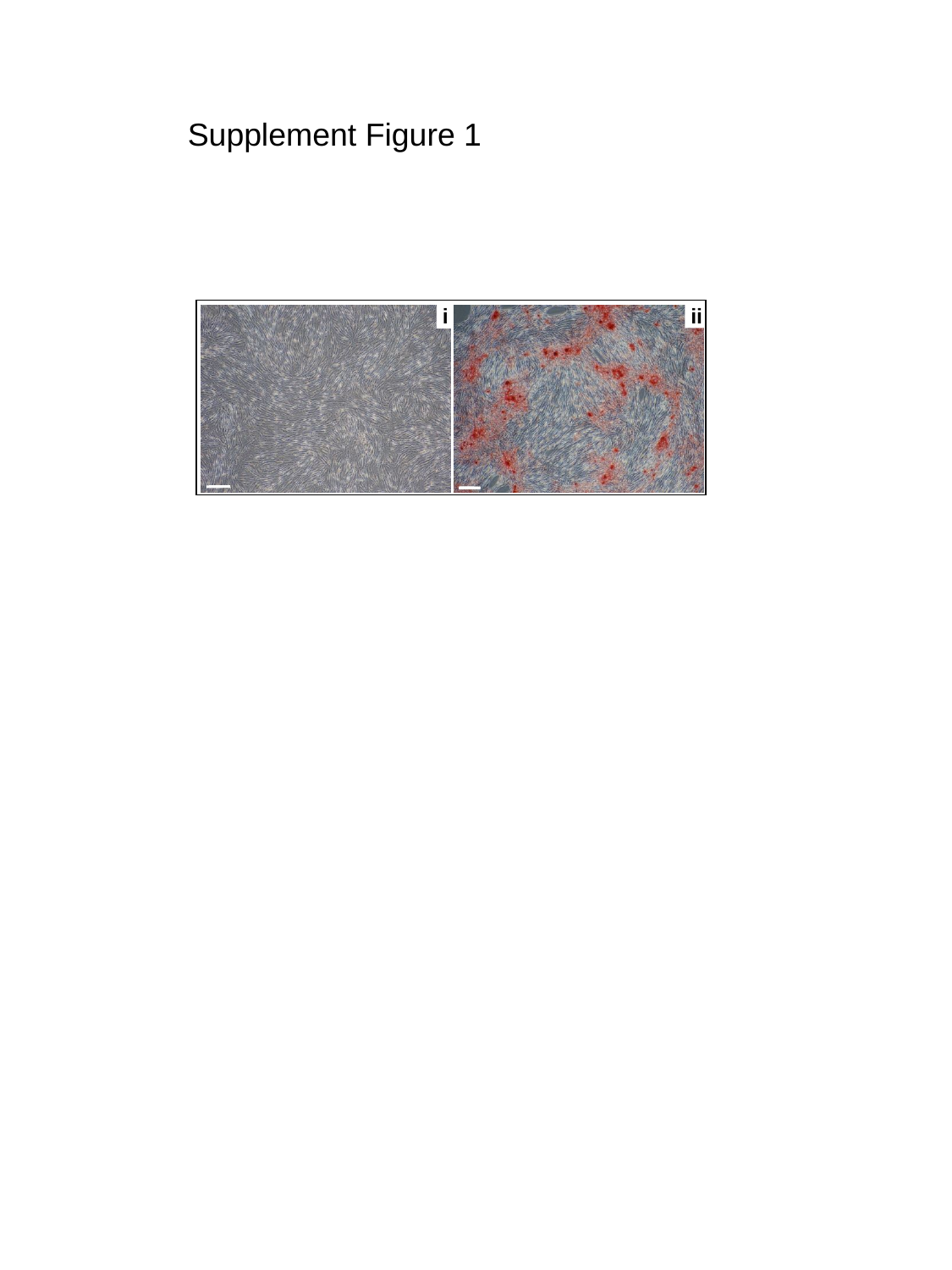

Supplement Figure 1
 ii
 i

## Slide 2
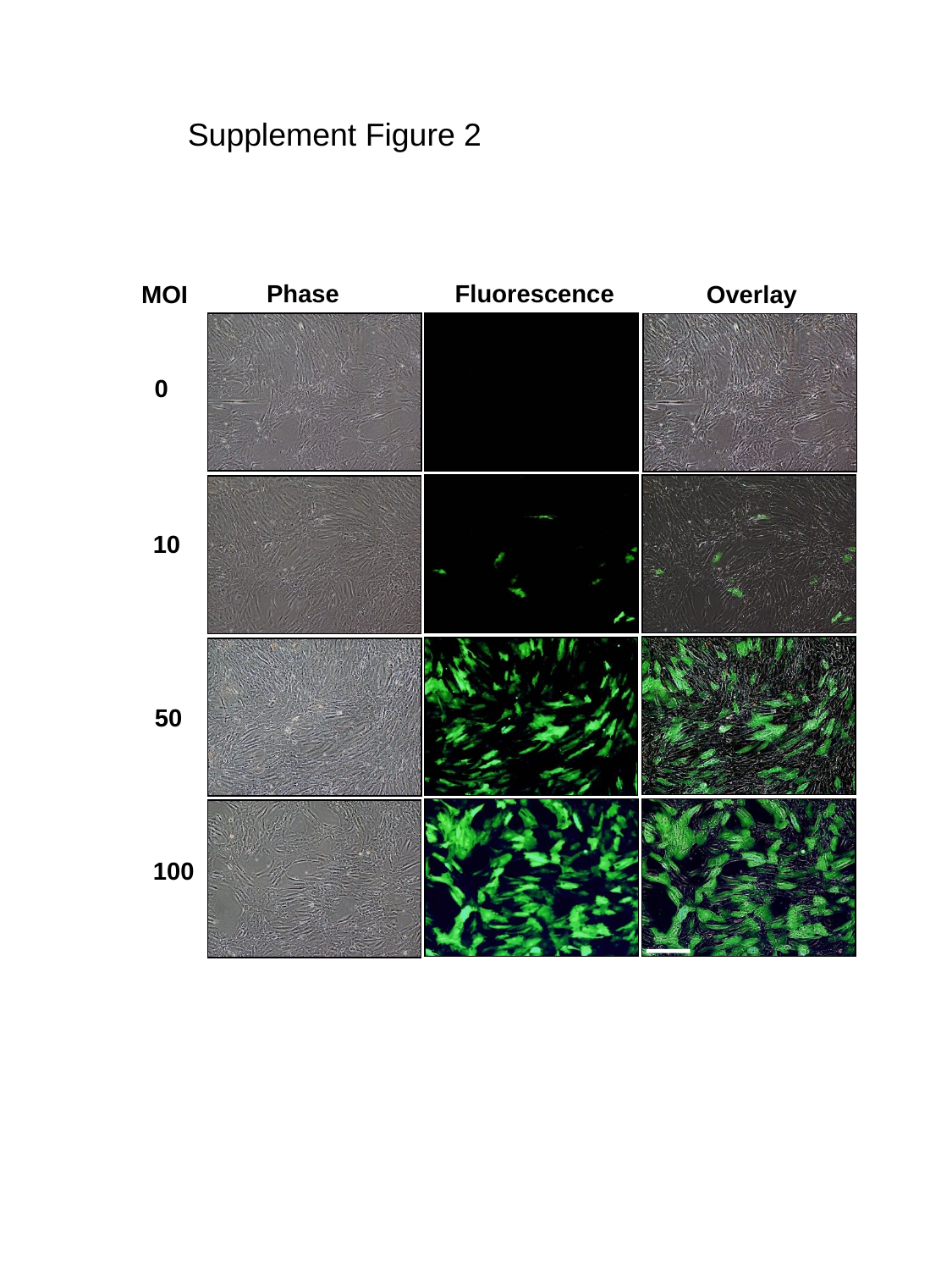

Supplement Figure 2
Phase
Fluorescence
MOI
Overlay
0
10
50
100

## Slide 3
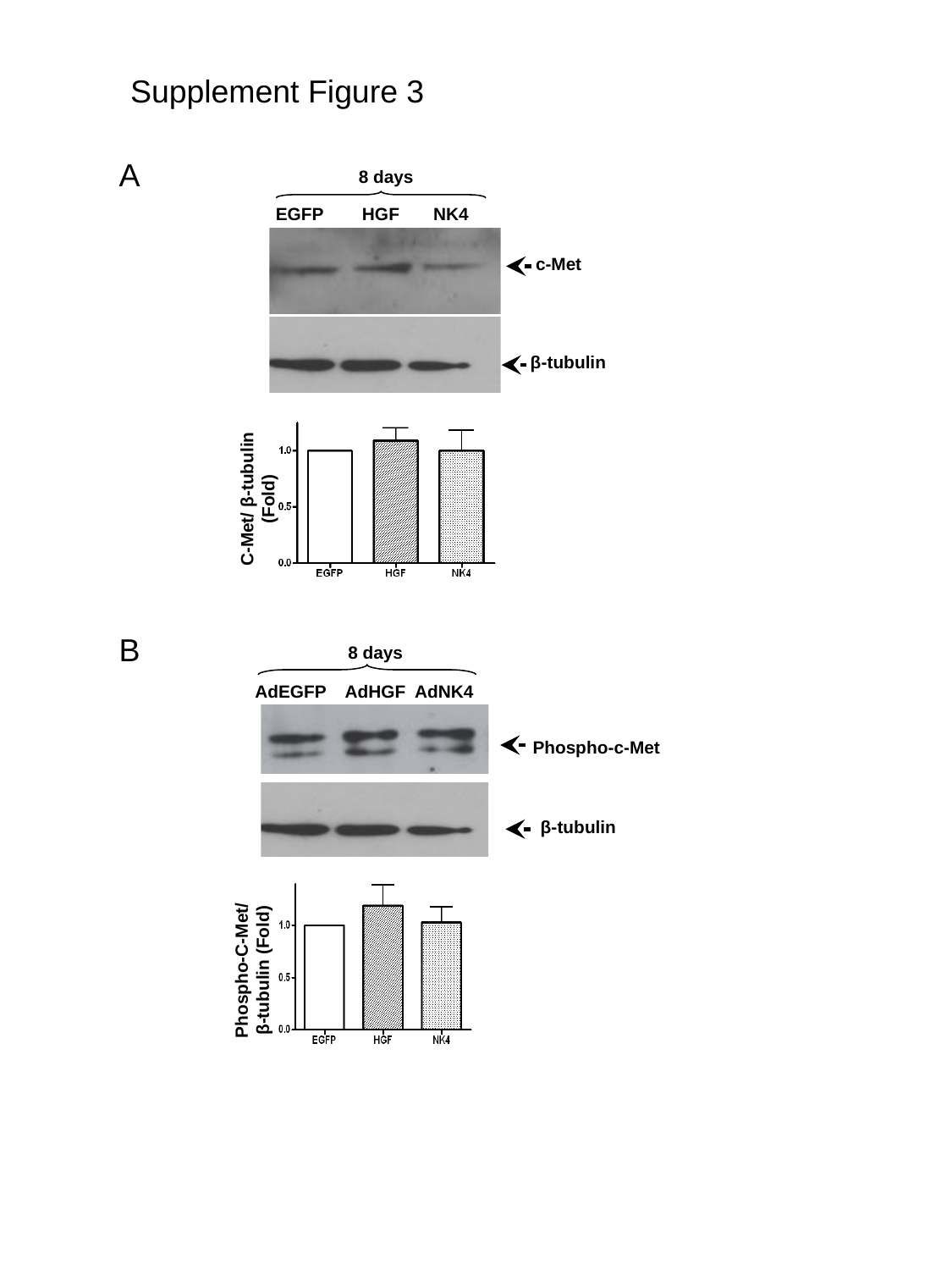

Supplement Figure 3
A
8 days
EGFP HGF NK4
c-Met
β-tubulin
C-Met/ β-tubulin (Fold)
B
8 days
AdEGFP AdHGF AdNK4
Phospho-c-Met
β-tubulin
Phospho-C-Met/ β-tubulin (Fold)
