# Supplementary material for: HGF/c-Met signalling promotes Notch3 activation and human vascular smooth muscle cell osteogenic differentiation in vitro
Source: Atherosclerosis. 2011 Dec;219(2):440–7. doi: 10.1016/j.atherosclerosis.2011.08.033 (PMC3925803; doi:10.1016/j.atherosclerosis.2011.08.033)
Supplement: Supplementary file 3 [file mmc3.ppt]

## Slide 1
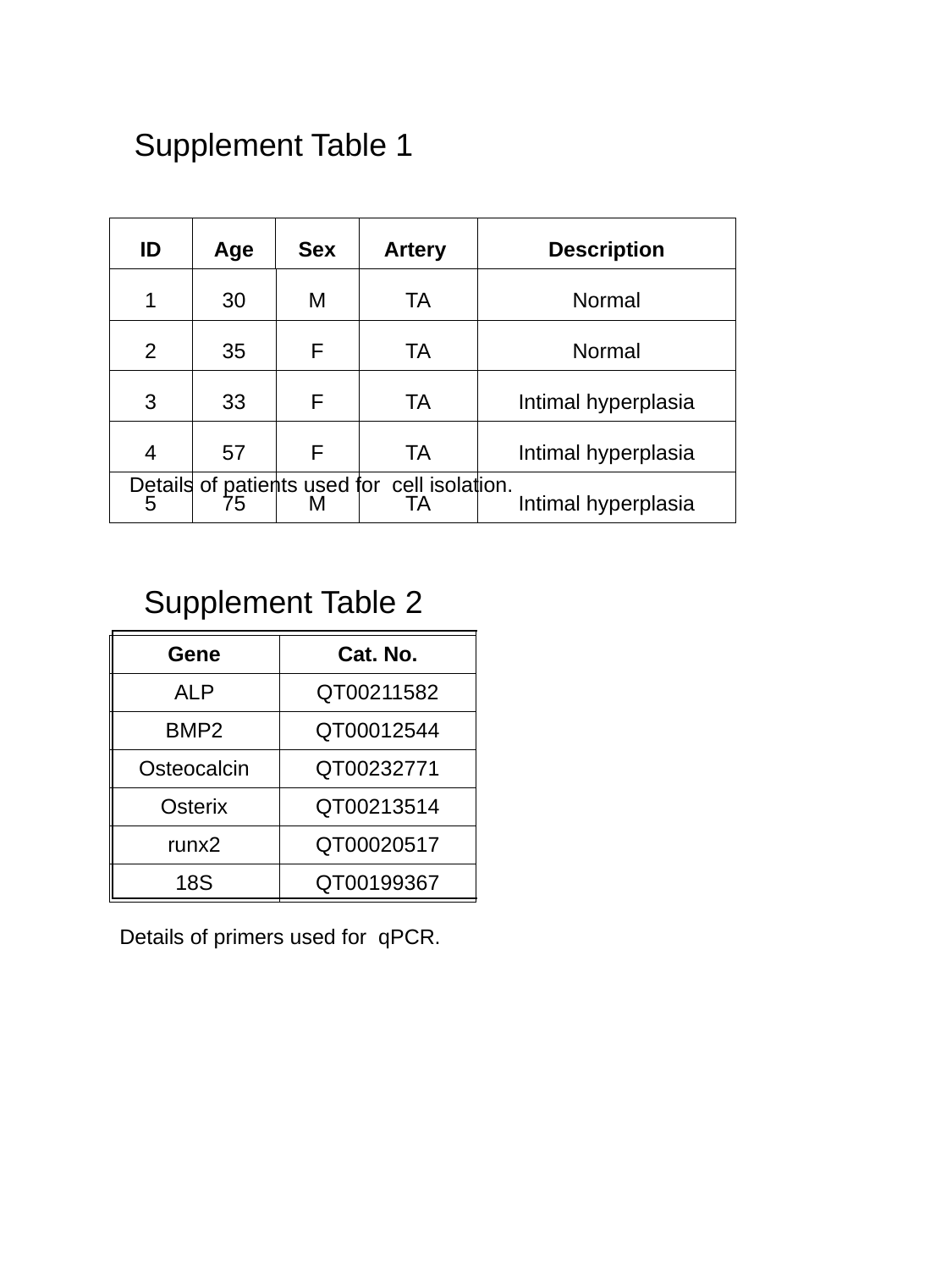

Supplement Table 1
| ID | Age | Sex | Artery | Description |
| --- | --- | --- | --- | --- |
| 1 | 30 | M | TA | Normal |
| 2 | 35 | F | TA | Normal |
| 3 | 33 | F | TA | Intimal hyperplasia |
| 4 | 57 | F | TA | Intimal hyperplasia |
| 5 | 75 | M | TA | Intimal hyperplasia |
Details of patients used for cell isolation.
Supplement Table 2
| |
| --- |
| Gene | Cat. No. |
| --- | --- |
| ALP | QT00211582 |
| BMP2 | QT00012544 |
| Osteocalcin | QT00232771 |
| Osterix | QT00213514 |
| runx2 | QT00020517 |
| 18S | QT00199367 |
Details of primers used for qPCR.
